# Development of Mini-Barcode Based on Chloroplast Genome and Its Application in Metabarcoding Molecular Identification of Chinese Medicinal Material Radix *Paeoniae Rubra* (Chishao)

**DOI:** 10.3389/fpls.2022.819822

**Published:** 2022-03-31

**Authors:** Xia Yang, Xiaolei Yu, Xiaoying Zhang, Hua Guo, Zhimei Xing, Liuwei Xu, Jia Wang, Yuyan Shen, Jie Yu, Pengfei Lv, Yuefei Wang, Mengyang Liu, Xiaoxuan Tian

**Affiliations:** ^1^State Key Laboratory of Component-Based Chinese Medicine, Tianjin University of Traditional Chinese Medicine, Tianjin, China; ^2^Tianjin Tongrentang Group Co., Ltd., Tianjin, China

**Keywords:** radix *Paeoniae Rubra*, chloroplast genome, mini-barcode, metabarcoding, Chinese patent medicine

## Abstract

Radix *Paeoniae Rubra* (Chishao), a typical multi-origin Chinese medicinal material, originates from the dried roots of *Paeonia lactiflora* or *P. veitchii*. The previous study suggested that these two commonly used Chishao showed variation in their chemical compositions and clinical efficacies. Therefore, accurate identification of different Chishao species was of great significance for the guide of clinical medication, and timely treatment of patients. In this study, the chloroplast genome sequences of *P. lactiflora* and *P. veitchii* were obtained by next-generation sequencing (NGS) technology, and then the hypervariable regions were selected to design two mini-barcode candidates for species identification. Combined with DNA metabarcoding technology, we performed qualitative and quantitative analysis on the artificially mixed samples of *P. lactiflora* and *P. veitchii* and evaluated the identification ability of these mini-barcode candidates. Furtherly, the mini-barcode with good performance was applied to distinguish the Chinese patent medicine “cerebral thrombosis tablets” containing Chishao. The results indicated that the chloroplast genomes of *P. lactiflora* and *P. veitchii* were 152,750 and 152,527 bp, respectively. As published previously, they exhibited a typical quadripartite structure including a large single-copy region (LSC), a small single-copy region (SSC) and a pair of inverted repeat regions (IRs). The nucleotide polymorphism analysis revealed seven variable protein-coding regions as *petL*, *psaI*, *psbJ*, *rpl16*, *ycf1b*, *psaC*, and *ndhF*, and two mini-barcodes were developed from *ycf1b* and *ndhF* respectively. The result suggested that both two mini-barcodes performed well distinguishing *P. lactiflora* from *P. veitchii*. Besides, *P. lactiflora* was the only raw material of Chishao in all collected “cerebral thrombosis tablets” samples. In general, this study has established a method to realize the qualitative and quantitative identification of Chishao as multi-origin Chinese medicinal materials, which can be applied to Chinese patent medicines containing Chishao.

## Introduction

Chinese medicinal materials are used as raw materials for various Chinese medicinal products, generally derived from animals, plants and minerals. One medicinal herb that originated from more than one species of plants, is called multi-origin Chinese herbal medicines in the Chinese Pharmacopeia, which occupies a large proportion of Chinese medicine products. However, studies have shown that the chemical components of multi-origin Chinese medicines are frequently different due to the utilization of different species. For example, phenolic pigments and essential oils represent the main chemical components of *Curcuma* (Yang et al., 2006), but their content varies amongst different plant parts and different plant species.

Chishao, the dried roots of *Paeonia lactiflora* Pall or *P. veitchii* Lynch belonging to Ranunculaceae, possesses various medicinal properties. According to classical medical literature in China, Chishao is bitter in taste and slightly cold in nature, with the function of clearing heat, cooling the blood, eliminating silt, and relieving pain (National Pharmacopoeia Committee, 2015). Modern studies have demonstrated that Chishao has wide pharmacological activities, including anti-tumor, liver protection, anti-thrombotic, anti-inflammatory, cardiovascular, and cerebrovascular protection (Jin et al., 2012; Wang et al., 2012; Xu et al., 2014; Huang et al., 2016; Lin et al., 2016; Xie et al., 2017; Liu et al., 2019; Tu et al., 2019). Currently, over 150 chemical components have been isolated and identified from Chishao (Liang et al., 2013). Besides monoterpene glycoside as the primary active compound, it also contains phenolic acids, triterpenes, flavonoids, volatile oil, etc., (Tanaka et al., 2000; Parker et al., 2016; Jiang et al., 2020).

The results of qualitative and quantitative chemical analyses indicate that, although the chemical compositions of Chishao species are similar generally, the content of phenolic acids from two species is rather different (Xu et al., 2009). In addition, according to Parker et al. (2016), *P. lactiflora* has greater potential for anti-inflammatory, antiviral, antibacterial, and antioxidant therapeutic activities compared to *P. veitchii*. Therefore, the accurate identification of the original plant is one of the important factors for the quality control of Chishao and is highly effective for the safety of clinical applications. Currently, multilateral approaches including microscopic, chromatographic, and mass spectrometry analyses have been widely used to authenticate and distinguish Chishao in complex samples (Xu et al., 2009; Shi et al., 2016), but these methods are either time-consuming or complicated in operation. DNA barcoding, an established molecular technique for taxonomic identification in biodiversity studies and product authentication, has been applied to the authentication studies of Chishao (Kim et al., 2016). However, the DNA barcoding technology based on the traditional Sanger sequencing method is only suitable for objects of a single source (Frézal and Leblois, 2008), which is seen as helpless for traditional Chinese medicine products containing multiple herbal medicines. Therefore, the development of a molecular method for the authentication of Chishao within herbal products is urgently needed. DNA metabarcoding is an approach merging next-generation sequencing technologies with DNA barcoding, which enables sensitive high-throughput multispecies identification based on DNA extraction from complex Chinese medicine (Taberlet et al., 2012; Cheng et al., 2014).

As herbs DNA degradation occurs during the medicine processing, the DNA fragments in extraction are frequently too short to be the template for “standard” DNA barcoding amplification (Gao et al., 2019). DNA mini-barcodes with significantly reduced length barcode sequences (100–250 bp) have been introduced to improve PCR amplification success of samples with degraded DNA (Meusnier et al., 2008; Hajibabaei and McKenna, 2012; Raime and Remm, 2018). This approach seems suitable for the authentication of heavily processed materials with severe DNA degradation (Song et al., 2017). However, the chosen DNA region should have sufficient variable sites and DNA genetic stability, which could ensure identification efficiency (Leray et al., 2013; Govender et al., 2019).

The chloroplast genome has been widely accepted as a valuable source of data for understanding evolutionary biology (Xu et al., 2017). The nature of the chloroplast genome, including its small size, conserved structure and gene content, moderate evolutionary rate and variations among gene loci, has rendered this genome more useful in molecular evolutionary analyses and phylogenetic reconstruction than mitochondrial and nuclear genomes (Drouin et al., 2008; Daniell et al., 2016; Park et al., 2018; Biju et al., 2019; Li et al., 2020). In previous research, Yu et al. (2020) had developed specific DNA mini-barcode from chloroplast genomes, and integrated them with DNA metabarcoding technology to differentiate *Senna* medicinal materials qualitatively and quantitatively.

In this study, we (1) sequenced, assembled, and annotated the complete chloroplast genome sequence of *P. lactiflora* and *P. veitchii* from our herbal materials, and compared them with those reported previously in terms of genome size, gene structure and guanine-cytosine (GC) content (Zhang et al., 2016; Samigullin et al., 2018; Lee et al., 2019; Gao et al., 2020), and then search for hypervariable regions in protein-coding sequences to design mini-barcodes; (2) to combine the mini-barcode with DNA metabarcoding method to examine whether the former could be used for the qualitative and quantitative analysis of *P. lactiflora* and *P. veitchii*; (3) take “cerebral thrombosis tablets” as an example to confirm the applicability of designed mini-barcode in proprietary Chinese medicines.

## Materials and Methods

### Sample Collection and Preparation

The standard products of *P. lactiflora and P. veitchii* were purchased from the National Drug Standard Substance Management Platform, China. *P. lactiflora* and *P. veitchii* were artificially mixed in different proportions to obtain seven experimental mixtures before extraction. The details of these mixed samples were shown in Supplementary Table 1. In addition, seven different batches of Chinese patent medicine “cerebral thrombosis tablets” containing Chishao were purchased from Tianjin Tong Ren Tang Group Co., Ltd., (lot number AP11023, AP11014, AP11024, AP11025, and AP11027) and Tonghua Xindongri Co., Ltd., (lot number 20190103 and 20190102). The details of samples information were shown in Supplementary Table 2.

### Genome DNA Extraction

Following the manufacturer’s protocol, the Plant Genomic DNA Kit (TIANGEN BIOTECH Co., Ltd., Beijing, China) was used to extract the genomic DNA from *P. lactiflora* and *P. veitchii*. 50–100 mg of ground powder was added to a centrifuge tube containing 700 μl of 65°C pre-warmed GP1 buffer (add mercaptoethanol to the pre-warmed GP1 to make a final concentration of 0.1% before the experiment) and vortexed for 15 s, and then incubated in water bath at 65°C during 20 min after which 700 μl of chloroform was added to each sample and mixed by vortexing for 15 s, and centrifuged at 12,000 rpm for 5 min. The supernatants were transferred to a new 1.5 ml centrifuge tube and 700 μl of GP2 buffer was added and briefly vortexed. The solution was transferred into the adsorption column CB3 (adsorption column was placed in the collection tube) and centrifuged at 12,000 rpm (∼13,400 × *g*) for 30 s. After the filtrate in the collection tube was discarded, the adsorption column was returned to the collection tube. The 500 μl of GD buffer was added to the adsorption column CB3, centrifuged at 12,000 rpm (∼13,400 × *g*) for 30 s. After the filtrate was discarded, we added 600 μl of PW buffer to adsorption column CB3, centrifuge at 12,000 rpm (∼13,400 × *g*) for 30 s, discarded the filtrate (this step was repeated two times), and then placed the adsorption column back into the collection tubes. Centrifuge for 2 min at 12,000 rpm (∼13,400 × *g*) and discard the filtrate. The adsorption column CB3 was placed at room temperature for a few minutes to thoroughly dry the residual wash liquid in the adsorbent material. The adsorption column CB3 was transferred to a new centrifuge tube, and 50–200 μl of TE buffer was added to the middle of the adsorption membrane in suspension, kept at room temperature for 2 min, and centrifuged at 12,000 rpm (∼13,400 × *g*) for 2 min to obtain DNA solution. DNA extractions were carried out in a dedicated pre-PCR laboratory, and PCR mixes were set up in a dedicated no-DNA laboratory to minimize the risk of contamination. The laboratory and the equipment were UV sterilized. Genomic DNA concentrations were determined by the measurement of optical density at 260 nm (OD260) using a NanoDrop™ spectrophotometer (Thermo Scientific, United States) and the purity was evaluated by measurement of the OD260/OD280 ratio. The sequencing library was generated using Truseq Nano DNA HT Sample preparation Kit (Illumina United States) following the manufacturer’s recommendations. The library was sequenced on Illumina HiSeq X Ten platform (Novogene, Nanjing, China) and 150 bp paired-end reads were generated.

### Chloroplast Genome Assembly and Annotation

Clean reads were obtained from raw reads by removing adapter sequences, reads containing N (N denotes not determined base information), and low-quality reads (reads with Qphred ≤ 20 bases accounting for more than 50% of the entire read length). The clean reads 6,021,633 and 6,072,563 were generated, respectively, from *P. lactiflora* and *P. veitchii* genomic DNA and used in the subsequent analysis. The chloroplast genome sequence of *P. rockii* (NCBI accession number: NC_037772) was used as the reference sequence, and clean reads of *P. lactiflora and P. veitchii* were mapped to the reference genome using Bowtie 2 (Langmead and Salzberg, 2012) software under the default parameters. And then we selected a consensus sequence with a deeper coverage (1–2 kb) as the seed sequence. Subsequently, the clean reads of *P. lactiflora* and *P. veitchii* were *de novo* assembled separately using NOVOPlasty 3.7.2 (Dierckxsens et al., 2017) software. The parameters were set as: insert size = 350, read length = 150, type = chloro, gene range = 10,000–20,000, k-mer = 39. The chloroplast genome sequences were annotated by the online program GeSeq (Tillich et al., 2017), followed by manual corrections of the start and stop codons based on the previously published chloroplast genome of *Paeonia*. The boundaries of tRNA genes were further detected using tRNAscan-SE online search serve (Lowe and Chan, 2016). Finally, the chloroplast genome maps were drawn using OGDRAW (Lohse et al., 2007).

Simple sequence repeats (SSRs) were identified by MISA with minimum repeat numbers of 10, 5, 4, 3, 3, and 3 for mono-, di-, tri-, tetra-, penta-, and hexanucleotides, respectively. Repeat sequences (forward, reverse, palindromic, and complement repeats) in the two Chishao species were analyzed through REPuter^1^, with the following setting: sequence identity was 90%, the Hamming distance was 3, and the minimum repeat size was 30 bp. Then, the contraction and expansion of the IR regions at the junctions of four main parts (LSC/IRb/SSC/IRa) of the chloroplast genomes were inspected and plotted with IRscope.

Two Chishao chloroplast genomes were compared with the previously reported chloroplast genomes in terms of genome size, genome structure and GC content. In addition, sequence alignment of amplicon regions (trimming primers) was performed using Geneious software. The phylogenetic analysis of these genes was constructed in MEGA-X software using the neighbor-joining (NJ) cluster algorithm. Evolutionary distances were calculated using the Kimura 2-parameter model and 1,000 replicate bootstrap tests, and *P. jishanensis* T. Hong and W. Z. Zhao was chosen as the outgroup (Sang and Zhang, 1999; Pan et al., 2007).

### Development of Specific DNA Mini-Barcode

We failed to find suitable conserved regions within long non-protein coding region, on the other hand, the short non-protein coding fragments did not contain enough information for species discrimination. Therefore, the protein-coding region was finally chosen to design primers for molecular identification. In our experiment, the protein-coding genes of the two chloroplast genomes were, respectively, extracted and aligned using PhyloSuite_v1.1.15 (Zhang et al., 2020). A sliding window analysis was performed using DNASP software version 6.11.01 (Rozas et al., 2017) to evaluate nucleotide diversity (Pi) and select hypervariable regions as candidates for mini-barcode development with the following parameters: window length 100 bp and step size 25 bp. Then, the primers were designed with Primer Premier 6.0. Finally, we used Oligo7.0 (Rychlik, 2007) to evaluate the characteristics of the selected primers. Primers that were likely to have hairpin structures, primer dimers, or excessive annealing temperature were abandoned.

### Validation of Mini-Barcode on Experimental Herb Mixtures and Chinese Patent Medicine

Genomic DNA was extracted from the mixtures of *P. lactiflora* and *P. veitchii* and from Chinese patent medicine samples using the Plant Genomic DNA Kit (TIANGEN BIOTECH Co., Ltd., Beijing, China) as described above. PCR assays were carried out in 25 μl reaction volumes containing 12.5 μl 2 × Gflex buffer (Takara Dalian, China), 0.5 μl Tks Gflex DNA Polymerase (Takara Dalian, China), 2 μl DNA template, 0.5 μl of each tagged primer, and 9 μl ddH_2_O, and with the following program settings: initial denaturing at 98°C for 1 min, followed by 30 cycles of 98°C for 10 s, 55°C for 15 s, 68°C for 30 s with a final extension at 68°C for 5 min. The negative PCR controls were analyzed in parallel with the samples to monitor for possible contamination. PCR products (including negative controls) were separated on 2% agarose gels and stained by ethidium bromide at 110 V for 30 min to determine the length of the amplified product fragments and estimate the concentration. Subsequently, to reduce the cost of independent library construction for each sample, the PCR products were mixed in equimolar concentrations for NGS PCR-free library construction. The library was sequenced on an Illumina HiSeq X Ten platform (Novogene, Nanjing, China), generating PE150 reads. To distinguish amplicons originating from the different samples, barcode oligonucleotides of 8 bp in length were ligated to each side of the primers, as shown in Supplementary Tables 3, 4.

The fastq-multx (Aronesty, 2013) was used to split the generated data into each sample. Primer sequences were trimmed by Cutadapt (Kechin et al., 2017). Subsequently, DADA2 (Callahan et al., 2016) package was used to control the sequence quality, and the parameters are set as maxEE = c (2, 2), minLen = 110, truncLen = c (120, 120). The base quality decreases when the sequence length was greater than 120 bp, so this part of the sequence was truncated (Supplementary Figure 1). Guessing error models, dereplication, denoising of paired-end reads, chimera removal, and creation of Amplicon Sequence Variants (ASVs) were performed with the DADA2 R package. ASV sequences with a total number of reads coverage less than 1,000 across all samples, which may be caused by sequencing errors or PCR mismatches, were filtered out (Caporaso et al., 2011). Then, the obtained ASV representative sequences were aligned with the *P. lactiflora* and *P. veitchii* reference region for species identification.

## Results

### Chloroplast Genome Characteristics of Chishao

**FIGURE 1:**
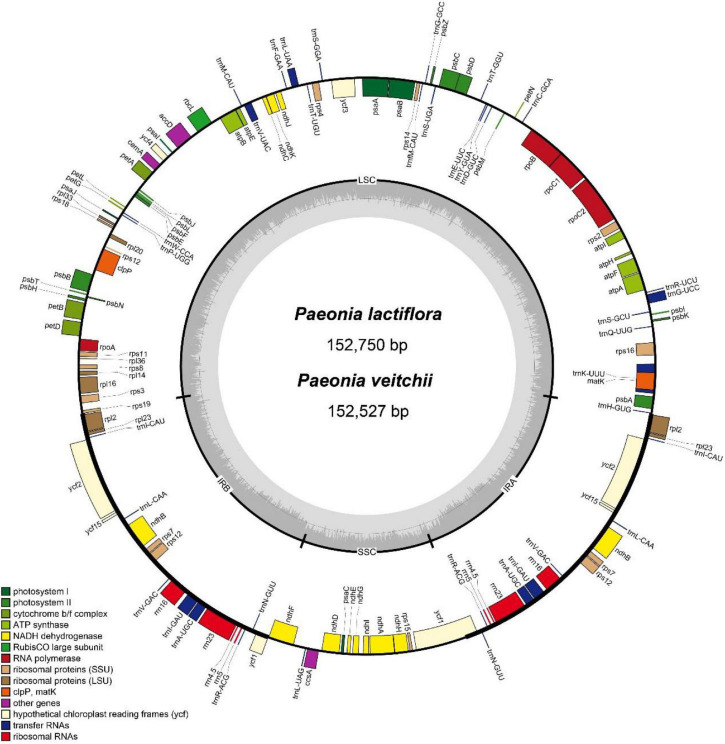
Gene map of the complete chloroplast genomes of *Paeonia lactiflora* and *Paeonia veitchii* in this study. Genes on the inside of the circle are transcribed clockwise, whereas those outside are transcribed counter-clockwise. The darker and lighter gray in the inner circle corresponds to GC content and AT content respectively.

For *P. lactiflora* and *P. veitchii*, the total length of the circular chloroplast genome was determined to be 152,750 and 152,527 bp, containing an SSC region of 16,971 or 17,006 bp, and an LSC region of 84,417 and 84,243 bp, separated by 2 copies of an IR of 25,680 or 25,639 bp, respectively (Figure1). The GC contents of two chloroplast genomes were 38.4%, with IR regions having higher GC content (43.1% and 43.2% in *P. lactiflora* and *P. veitchii*, respectively) than LSC (36.7% in both species) and SSC regions (32.7% in both species). Both chloroplast genomes encoded 111 predicted genes, as 77 protein-coding genes, 4 transfer RNA (tRNA) genes, and 30 ribosomal RNA (rRNA) genes. Nineteen genes were duplicated in the IR regions, including 8 protein-coding genes, 7 tRNA genes and 4 rRNA genes. There were 17 intron-containing genes, 15 of which contained a single intron, while two genes contained two introns. Information of these genes were shown in Supplementary Table 5.

**FIGURE 2:**
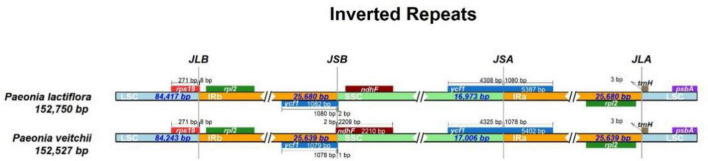
Comparison of the LSC, IR, and SSC junction positions between *Paeonia lactiflora* and *Paeonia veitchii.*

The IR/LSC and IR/SSC borders of the *P. lactiflora* and *P. veitchii* chloroplast genomes were compared (Figure 2). In two chloroplast genomes, the *rps19* gene crossed the LSC and IRb regions, with 8 bp extension into the IRb region. The *ndhF* gene crossed the IRb/SSC boundary in *P. veitchii* and formed a partial overlapping region with the *ycf1* pseudogenes, however, in *P. lactiflora*, the *ndhF* gene was totally located in the SSC region. Two chloroplast genomes had similar SSC/IRa and IRa/LSC borders: the *ycf1* gene crosses the SSC/IRa boundary, most of which was located in the SSC region. On the other hand, the *rpl2* gene was in the IRa region and the *psbA* gene in the LSC region, and the *trnH* gene spanned the junction between IRa and LSC regions.

As molecular marker candidates for analyzing the genetic diversity and population structure, SSRs were made up of abundant tandem repeat sequences consisting of 1–6 bp units. We have identified 57 and 69 SSRs sites in the chloroplast genomes of *P. lactiflora* and *P. veitchii*, respectively. All these SSRs found were shorter than 18 bp. The number of mononucleotide SSRs sites was the richest (35 and 42 in *P. lactiflora* and *P. veitchii*, respectively), and most of them were A/T (Table 1). This result was congruent with the previous observation that chloroplast genome SSRs were generally composed of A/T, and rarely C/G (Kuang et al., 2011). The second abundant SSRs were dinucleotide repeats, followed by trinucleotide repeats, while hexanucleotide repeats were not found.

**TABLE 1:** Types and numbers of SSRs in the chloroplast genomes of *Paeonia lactiflora* and *Paeonia veitchii.*

		Species	
		
SSR Type	Repeat unit	*Paeonia lactiflora*	*Paeonia veitchii*
Mono	A/T	35	42
	C/G	0	2
Di	AG/CT	1	1
	AT/AT	10	12
Tri	AAT/ATT	7	7
Tetra	AAAC/GTTT	1	1
	AAAT/ATTT	2	2
	AGAT/ATCT	1	1
Penta	AAGAT/ATCTT	0	1
Hexa			
Total		57	69

**FIGURE 3:**
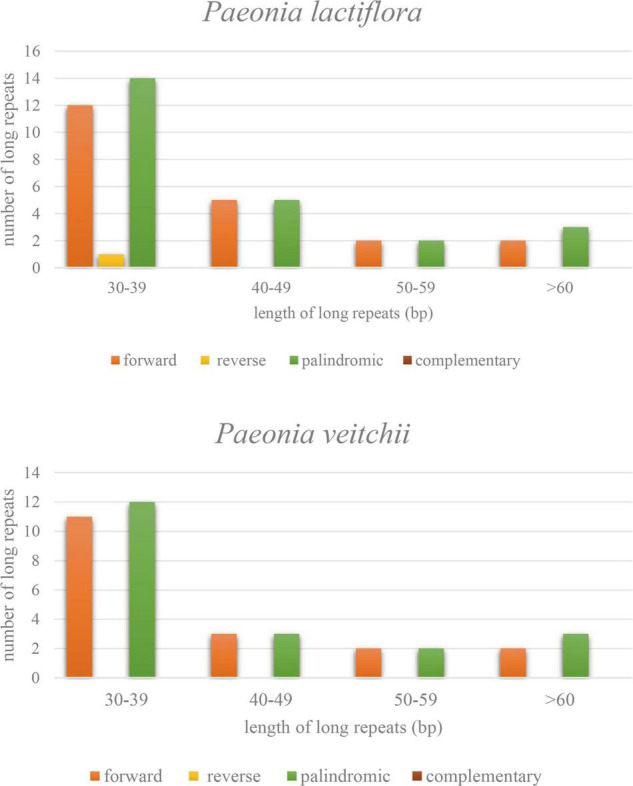
Types and numbers of long repeat sequences in the chloroplast genomes of *Paeonia lactiflora* and *Paeonia veitchii*.

Three types of long repeat sequences, namely forward repeat, reverse repeat and palindromic repeat, were found in the *P. lactiflora* chloroplast genome, while only the first and third ones were found in *P. veitchii*. The majority of these repeats were mainly forward and palindromic types with lengths mainly in the range of 30–39 bp in both Chishao species (Figure 3).

Furthermore, all published five *P. lactiflora* and three *P. veitchii* complete chloroplast genomes in GenBank database were simultaneously taken into consideration for comparative analyses (Zhang et al., 2016; Samigullin et al., 2018; Lee et al., 2019; Gao et al., 2020; Tables 2, 3). The result showed that the two Chishao chloroplast genomes exhibited high similarity with previously published data in genome size and structure, gene number, as well as GC content.

**TABLE 2:** A summary of the complete chloroplast genome, IR, LSC and SSC length (bp) and GC content of *Paeonia lactiflora*.

		Complete chloroplast genome		IR		LSC		SSC	
					
GenBank	Data resource	Size (bp)	GC content (%)	IRs size (bp)	GC content (%)	LSC size (bp)	GC content (%)	SSC size (bp)	GC content (%)
MW762595	This study	152,750	38.4	25,680	43.1	84,414	36.7	16,956	32.7
NC_040983	Samigullin et al., 2018	152,747	38.4	25,651	43.1	84,412	36.7	17,033	32.7
MK860971	Lee et al., 2019	152,731	38.4	25,680	43.1	84,402	36.7	16,969	32.7
MN868412	Gao et al., 2020	153,405	38.4	26,048	43.0	84,285	36.7	16,969	32.7
MG897127	Samigullin et al., 2018	152,747	38.4	25,651	43.1	84,412	36.7	17,033	32.7

**TABLE 3:** A summary of the complete chloroplast genome, IR, LSC and SSC length (bp) and GC content of *Paeonia veitchii*.

GenBank	Data resource	Complete chloroplast genome		IR		LSC		SSC	
		Size (bp)	GC content (%)	IRs size (bp)	GC content (%)	LSC size (bp)	GC content (%)	SSC size (bp)	GC content (%)
MW762596	This study	152,527	38.4	25,639	43.2	84,240	36.7	17,006	32.7
NC_032401	Zhang et al., 2016	152,682	38.4	25,653	43.1	84,398	36.7	16,978	32.7
KT894821	Zhang et al., 2016	152,682	38.4	25,653	43.1	84,398	36.7	16,978	32.7

### Design of Mini-Barcode for Species Identification

**FIGURE 4:**
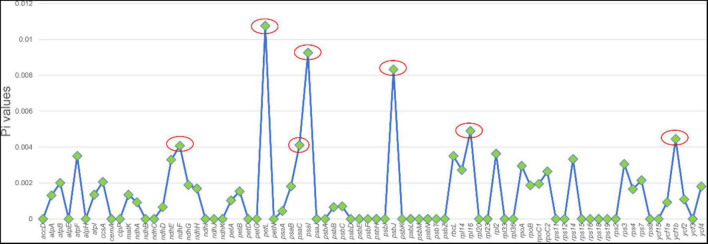
Nucleotide diversity of different regions between *Paeonia lactiflora* and *Paeonia veitchii* chloroplast genome in this study. The x-axis shows chloroplast genome regions and the y-axis shows nucleotide diversity.

Highly variable DNA regions of chloroplast genomes could be used to distinguish between closely related species (Jiao et al., 2019). In this study, a total of 77 protein-coding genes shared between two Chishao species were used to estimate nucleotide diversity. Nucleotide diversity values (Pi) varied from 0 to 0.01075, with an average of 0.00135, indicating a mild divergence between two genomes in general. However, as shown in Figure 4, seven loci showing remarkably higher Pi values (>0.004), as *pstL* (0.01075), *psaI* (0.00926), *psbJ* (0.00833), *rpll* (0.0049), *ycf1b* (0.0046), *pasC* (0.00412), and *ndhF* (0.00408), were suitable for mini-barcode design. Considering the limitation of barcode length, primer conservation, primer physical and chemical properties, two primer pairs were successfully designed on the *ycf1b* and *ndhF* regions finally (Table 4). The two amplicons of CS-*ycf1b* and CS-*ndhF* were 223 and 250 bp in length respectively, and each had four variable positions.

**TABLE 4:** Primer information of two mini-barcodes.

Primer pair name	CS-*ycf1b*	CS-*ndhF*
Forward primer sequence 5′–3′	CCTCTACATAATCCGA	GCGTTATCTTTCTTCC
Reverse primer sequence 5′–3′	CAATCAGATTTTCGTCG	ACAGCAGGATTAACTG
Amplicon size (bp)	223	250
Variable sites of mini-barcode	4	4
Length of sequence excluding primers (bp)	190	218
GC% (Forward/Reverse)	43.8/41.2	43.8/43.8
Tm (°C) (Forward/Reverse)	44.8/48.1	46/46.9

**FIGURE 5:**
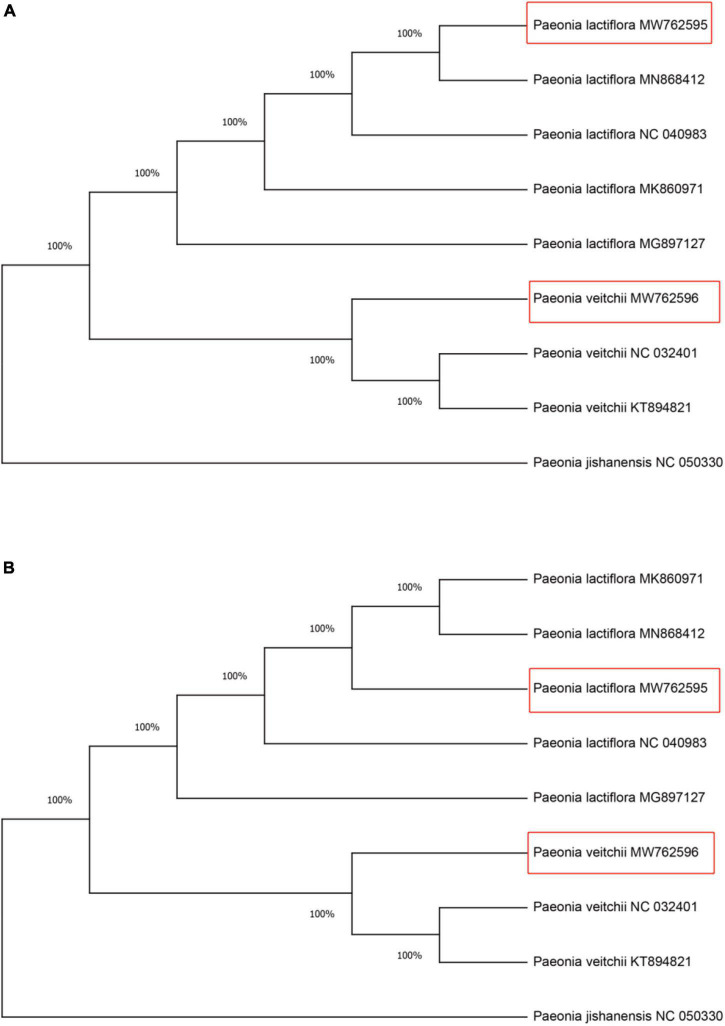
Neighbor-Joining (NJ) tree of the Chishao species based on primers CS-*ycf1b* and CS-*ndhF* amplified sequences. **(A)** Primer CS*-ycf1b*; **(B)** Primer CS*-ndhF.* Numbers beside nodes indicate statistical support for individual clades (percent), based on 1,000 bootstrap replicates of the data. Red boxes represent the assembled and annotated chloroplast genomes of *Paeonia lactiflora* and *Paeonia veitchii* in this study. *Paeonia jishanensis* was used as the outgroup. The GenBank accession numbers were listed following the species name.

Within all available chloroplast genome sequences of two Chishao species, CS-*ycf1b* and CS-*ndhF* amplicon regions (primers trimmed) were extracted in Geneious. Neighbor Joining (NJ) was applied for constructing gene trees (Molloy and Warnow, 2018). As shown in Figure 5, all individuals from the same species clustered together and two species formed monophyletic clades with 100% bootstrap support based on either marker gene tree.

### Qualitative and Quantitative Analysis of DNA Mini-Barcode

As the validation of our newly designed primer pairs CS-*ycf1b* and CS-*ndhF*, all samples were successfully amplified in seven experimental mixtures (Supplementary Figure 2). All PCR amplicons were sequenced, generating a total of 14,852,184 clean reads after filtering out adaptor sequences, ambiguous reads and low-quality reads. For all three experimental repeats, based on similarity, the amplification products of CS*-ycf1b* were clustered into 6 ASVs, while three of them were assigned as *P. lactiflora* (yASV2, yASV3 and yASV6) and three as *P. veitchii* (yASV1, yASV4 and yASV5). The amplification products of CS-*ndhF* were clustered into 5 ASVS, and one of them was assigned as *P. lactiflora* (nASV1) and the others as *P. veitchii* (Supplementary Table 6).

**FIGURE 6:**
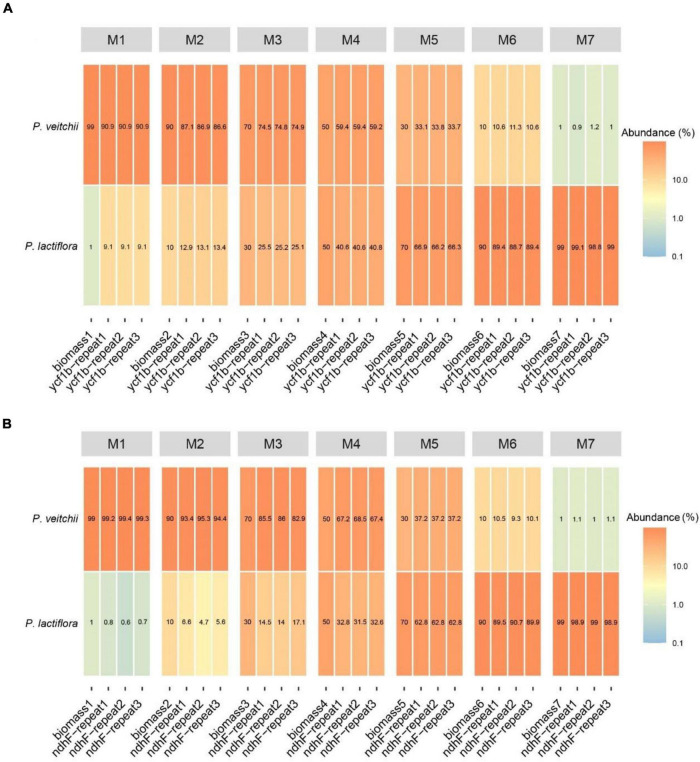
Heatmap of reads abundance of two pairs of different primers in seven experimental mixtures **(A)** Primer CS*-ycf1b*; **(B)** Primer CS*-ndhF*.

**FIGURE 7:**
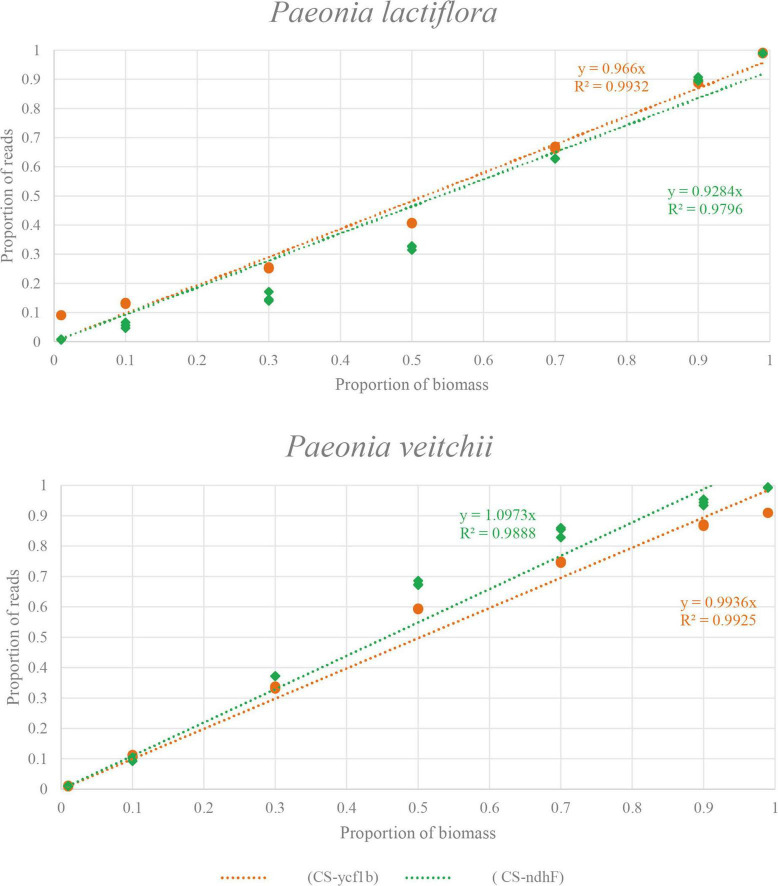
The relationship between biomass and read counts of the products amplified by two primer pairs (CS*-ycf1b* and CS*-ndhF*) in two species. X-axis, the proportion of biomass; Y-axis, the proportion of reads.

Subsequently, we performed the correlation analysis between the species biomass proportion and species reads proportion in seven mixtures to assess the quantitative capacity of the mini-barcodes. As shown in Figure 6, results were repeatable between replicates from the same DNA extractions for both Chishao species, and a positive correlation between the sequence read counts and real proportion of individuals was illustrated. The species reads proportion for primer CS*-ycf1b* was more closely related to the species biomass proportion relative to primer CS*-ndhF*. This implied that the mini-barcode of primer CS*-ycf1* seems have relatively better quantitative ability (*P. lactiflora*: R^2^ = 0.9932; *P. veitchii*: R^2^ = 0.9925) (Figure 7).

### Application of Mini-Barcode in Chinese Patent Medicine

**FIGURE 8:**
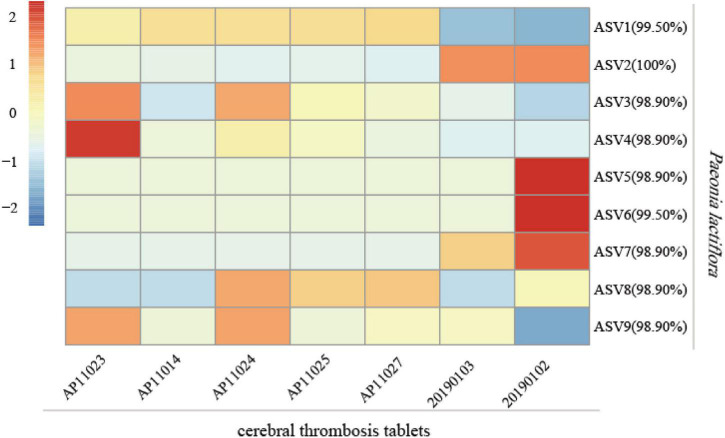
Analysis of the species of Chishao in 7 batches of Chinese patent medicine “cerebral thrombosis tablets.” The read counts of sequencing data were normalized by Z-Score normalization. The formula for Z-Score was (X- mu)/sigma, where X was the read counts of each sample, mu and sigma were the mean value and standard deviation, respectively. The legend indicated the Z-score for each sample. The percentage besides amplicon sequence variants (ASV) number in the figure denoted the similarity between the representative sequences of ASV and the reference sequence.

To examine the practicability of our mini-barcode in Chinese patent medicine, DNA was extracted from seven batches of “cerebral thrombosis tablets” and was then subjected to PCR amplification by using specific primers CS*-ycf1*. We found that all samples showed successful PCR amplification (Supplementary Figure 3). The PCR amplicons obtained were sequenced and Illumina sequencing generated a total of 2,359,378 high-quality filtered reads, which were clustered into 9 ASVs, and all of which were identified as *P. lactiflora* (Figure 8). Interestingly, the composition of ASVs varied much among 7 samples, and indicated the subspecies level biodiversity of Chishao, which may also affect medicine effection and need more exploration in future.

## Discussion

Limit of detection (LOD), is defined as the minimum amount or concentration of the analyte in the tested sample, which can be reliably detected. The main findings from our assessment of detection sensitivity and accuracy associated with the mixture of *P. lactiflora* and *P. veitchii* constructed by the metabarcoding experiment were that we could detect species with biomass percentages as low as 1% of the total sample mass. Given that the lowest theoretical detection limits were not tested in our study, we may be able to detect Chishao species with less biomass than we reported.

Besides, limit of quantification (LOQ) is defined as the lowest amount or concentration of the analyte in the tested sample, which can be quantitatively determined with an acceptable degree of accuracy and precision. In our research, quantitative data obtained for the seven experimental mixtures showed a linear relationship between the sequence read counts and real biomass proportion of individuals in the 100 mg of sample mixtures. In general, the primer CS-*ycf1* exhibited better correlation coefficient than primer CS-*ndhF* (*P. lactiflora*: R^2^ = 0.9796; *P. veitchii*: R^2^ = 0.9888).

It’s worth noting that, the primer CS*-ndhF* was found to have an amplification bias toward *P. veitchii* (e.g., M3 and M4). On the other hand, for primer pairs CS-*ycf1*, the quantified result of M1 showed quite high variation between theoretic value and measure value, although the tested value of M7 was close to the theoretic value, and for CS-*ndhF*, the tested values for M7 and M1 were both close to the theoretical values, indicating there should be no error in the preparation of given species biomass in our experiment. In addition, the sequencing results of the three replicates were highly reproducible, which showed good reliability of the experimental results. Our potential explanation for the observed proportion bias was that many factors operating during PCR amplification and sequencing, as well as the processing of metabarcoding data (chimeric sequences, contaminants, and clustering algorithms) can alter the correspondence between the percentage of reads retrieved and the species abundance (Elbrecht and Leese, 2015; Piñol et al., 2015; Matesanz et al., 2019).

The manufacturing and processing of Chinese medicine products (e.g., cooking, high pressure, pH modification, grinding or drying) could result in varying degrees of DNA degradation, which may affect the quantity and quality of DNA extracted from these products and reduce amplification efficiency. One of the current progress in identifying plant taxa in DNA degraded samples was the use of mini-barcodes (Shokralla et al., 2015). There were many types of Chinese patent medicines containing Chishao, such as tablets, capsules, and pills. Here we selected “cerebral thrombosis tablets” commonly used in the market for the validation experiment. “cerebral thrombosis tablets” contains Chishao and nine other ingredients as follows: *Carthamus tinctorius* (Honghua), *Angelica sinensis* (Danggui), *Whitmania pigra Whitman* (Shuizhi), *Prunus persica* (Taoren), *Ligusticum chuanxiong hort* (Chuanxiong), *Salvia miltiorrhiza* (Danshen), *Eupolyphaga seu Steleophaga* (Tubiechong), *Cornu Saigae Tataricae* powder (Lingyangjiao), *Bovis Calculus Artifactus* powder (Niuhuang). During the medicine preparation, Chishao was ground into a fine powder (80 mesh), mixed with other materials into granules, and finally pressed into tablets. In this study, our results revealed that the primer pair CS-*ycf1b* could successfully amplify the nucleotide signature region to identify the Chishao from tablets even if the Chishao was processed in various ways.

## Conclusion

In this study, based on the chloroplast genome data, the qualitative and quantitative identification of multi-origin Chinese herbal medicine Chishao was achieved by specific mini-barcode in combination with DNA metabarcoding method. Subsequently, the mini-barcode of primer CS-*ycf1b* was found useful for identifying processed Chinese patent medicine containing Chishao acquired in markets. Overall, we provided a research example for the identification of multi-origin herbal medicine within complex samples, which was exemplary for the subsequent application of the method.

## Data Availability Statement

The original contributions presented in the study are publicly available. This data can be found here: National Center for Biotechnology Information (NCBI) BioProject database under accession number PRJNA788072.

## Author Contributions

XT and XYu conceived the study. XYu and XZ performed the experiment. XYa assembled, annotated, and analyzed the plastomes. XYa drafted the manuscript. HG, ZX, and LX provided input to the manuscript. YW and XT revised the manuscript. JW, YS, JY, PL, and ML coordinated the related research works. All authors read and approved the final manuscript.

## Conflict of Interest

JW, YS, JY, and PL were employed by the company Tianjin Tongrentang Group Co., Ltd. The remaining authors declare that the research was conducted in the absence of any commercial or financial relationships that could be construed as a potential conflict of interest.

## Publisher’s Note

All claims expressed in this article are solely those of the authors and do not necessarily represent those of their affiliated organizations, or those of the publisher, the editors and the reviewers. Any product that may be evaluated in this article, or claim that may be made by its manufacturer, is not guaranteed or endorsed by the publisher.

## Footnotes

1
https://bibiserv.cebitec.uni-bielefeld.de/reputer

## Abbreviations

LSC: large single-copy region
IR: inverted repeats region
SSC: small single-copy region
ASV: amplicon sequence variant.
