# Corrigendum: Development of mini-barcode based on chloroplast genome and its application in metabarcoding molecular identification of Chinese medicinal material Radix *Paeoniae Rubra* (Chishao)

**DOI:** 10.3389/fpls.2024.1463138

**Published:** 2024-08-07

**Authors:** Xia Yang, Xiaolei Yu, Xiaoying Zhang, Hua Guo, Zhimei Xing, Liuwei Xu, Jia Wang, Yuyan Shen, Jie Yu, Pengfei Lv, Yuefei Wang, Mengyang Liu, Xiaoxuan Tian

**Affiliations:** ^1^ State Key Laboratory of Component-Based Chinese Medicine, Tianjin University of Traditional Chinese Medicine, Tianjin, China; ^2^ Tianjin Tongrentang Group Co., Ltd., Tianjin, China

**Keywords:** radix *Paeoniae Rubra*, chloroplast genome, mini-barcode, metabarcoding, Chinese patent medicine

**Error in Figure/Table**

In the published article, there was an error in **Table 1** Types and numbers of SSRs in the chloroplast genomes of *Paeonia lactiflora* and *Paeonia veitchii* as published. In the SSR tetranucleotide repeats for *Paeonia lactiflora* and *Paeonia veitchii*, the quantities for AAAC/GTTT, AAAT/ATTT, and AGAT/ATCT should be 1, 2, and 1, respectively. However, in the published version, it incorrectly shows AAAC/GTTT 121, with the other two fields left blank. The corrected **Table 1** Types and numbers of SSRs in the chloroplast genomes of *Paeonia lactiflora* and *Paeonia veitchii* and its caption appear below.

**Table 1:** Types and numbers of SSRs in the chloroplast genomes of *Paeonia lactiflora* and *Paeonia veitchii*.

		Species	
SSR Type	Repeat unit	*Paeonia lactiflora*	*Paeonia veitchii*
Mono Di Tri Tetra Penta Hexa Total	A/T C/G AG/CT AT/AT AAT/ATT AAAC/GTTT AAAT/ATTT AGAT/ATCT AAGAT/ATCTT	35 0 1 10 7 1 2 1 0 57	42 2 1 12 7 1 2 1 1 69

The authors apologize for this error and state that this does not change the scientific conclusions of the article in any way. The original article has been updated.

