# Supplementary material for: Development of Mini-Barcode Based on Chloroplast Genome and Its Application in Metabarcoding Molecular Identification of Chinese Medicinal Material Radix Paeoniae Rubra (Chishao)
Source: Front Plant Sci. 2022 Mar 31;13:819822. doi: 10.3389/fpls.2022.819822 (PMC9009180; doi:10.3389/fpls.2022.819822)
Supplement: Supplementary file 1 [file Data_Sheet_1.docx]

Supplementary Material

# Supplementary Tables

Table S1. Biomass of each species in the experimental mixture

| Experimental mixture | Species | | |
| --- | --- | --- | --- |
|  | *P. lactiflora* (mg) | *P. veitchii* (mg) | *P. lactiflora vs. P. veitchii* |
| 1 | 1 | 99 | 1:99 |
| 2 | 10 | 90 | 1:9 |
| 3 | 30 | 70 | 3:7 |
| 4 | 50 | 50 | 5:5 |
| 5 | 70 | 30 | 7:3 |
| 6 | 90 | 10 | 9:1 |
| 7 | 99 | 1 | 99:1 |

Table S2. Cerebral thrombosis tablets sample information

| Company | Voucher ID | Batch |
| --- | --- | --- |
| Tianjin Tong Ren Tang Group Co., Ltd  Tianjin Tong Ren Tang Group Co., Ltd  Tianjin Tong Ren Tang Group Co., Ltd  Tianjin Tong Ren Tang Group Co., Ltd  Tianjin Tong Ren Tang Group Co., Ltd  Tonghua Xindongri Co., Ltd  Tonghua Xindongri Co., Ltd | Zcy1  Zcy2  Zcy3  Zcy4  Zcy5  Zcy6  Zcy7 | AP11023  AP11014  AP11024  AP11025  AP11027  20190103  20190102 |

Table S3. Tag information of experimental mixture

| Primer name | Experimental mixture | Tag sequence | |
| --- | --- | --- | --- |
| CS-*ycf1b* | CSYC1 | Forward (5'-3') : GTGTGCAT | Reverse (5'-3') : GTGTGCAT |
|  | CSYC2 | Forward (5'-3') : CTCATGCT | Reverse (5'-3') : CTCATGCT |
|  | CSYC3 | Forward (5'-3') : CTGTCTCG | Reverse (5'-3') : CTGTCTCG |
|  | CSYC4 | Forward (5'-3') : CATGCTGT | Reverse (5'-3') : CATGCTGT |
|  | CSYC5 | Forward (5'-3') : CAGCTGAG | Reverse (5'-3') : CAGCTGAG |
|  | CSYC6 | Forward (5'-3') : CGTCATAC | Reverse (5'-3') : CGTCATAC |
|  | CSYC7 | Forward (5'-3') : GTCGCGTA | Reverse (5'-3') : GTCGCGTA |
| CS*-ndhF* | CSND1 | Forward (5'-3') : ACTAGCAG | Reverse (5'-3') : ACTAGCAG |
|  | CSND2 | Forward (5'-3') : ACGATGTC | Reverse (5'-3') : ACGATGTC |
|  | CSND3 | Forward (5'-3') : ACGCGAGA | Reverse (5'-3') : ACGCGAGA |
|  | CSND4 | Forward (5'-3') : AGTCTGCA | Reverse (5'-3') : AGTCTGCA |
|  | CSND5 | Forward (5'-3') : AGATCGAC | Reverse (5'-3') : AGATCGAC |
|  | CSND6 | Forward (5'-3') : AGCGTATG | Reverse (5'-3') : AGCGTATG |
|  | CSND7 | Forward (5'-3') : CTAGTAGC | Reverse (5'-3') : CTAGTAGC |

Table S4. Tag information of Chinese patent medicine samples

| Primer name | Voucher ID | Tag sequence | |
| --- | --- | --- | --- |
| CS*-ycf1b* | Zcy1 | Forward (5'-3') : GTGTGCAT | Reverse (5'-3') : GTGTGCAT |
|  | Zcy2 | Forward (5'-3') : CTCATGCT | Reverse (5'-3') : CTCATGCT |
|  | Zcy3 | Forward (5'-3') : CTGTCTCG | Reverse (5'-3') : CTGTCTCG |
|  | Zcy4 | Forward (5'-3') : CATGCTGT | Reverse (5'-3') : CATGCTGT |
|  | Zcy5 | Forward (5'-3') : CAGCTGAG | Reverse (5'-3') : CAGCTGAG |
|  | Zcy6 | Forward (5'-3') : AGTCTGCA | Reverse (5'-3') : AGTCTGCA |
|  | Zcy7 | Forward (5'-3') : AGATCGAC | Reverse (5'-3') : AGATCGAC |

Table S5. Chloroplast genome gene information of P. lactiflora and P. veitchii

| Category | Group | Genes |
| --- | --- | --- |
| Photosynthesis related genes | Rubisco | *rbcL* |
|  | Photosystem I | *psaA, psaB, psaC, psaI, psaJ* |
|  | Photosystem II | *psbA, psbB, psbT, psbK, psbI, psbH, psbM, psbN, psbD, psbC, psbZ, psbJ, psbL, psbE, psbF* |
|  | ATP synthase | *atpA, atpB, atpE, atpF^a^, atpH, atpI* |
|  | Cytochrome b/f complex | *petA, petB^a^, petD^a^, petN, petL, petG* |
|  | Cytochrome csynthesis | *ccsA* |
|  | NADPH dehydrogenase | *ndhA^a^, ndhB^a, c^ (×2), ndhC, ndhD, ndhE, ndhF, ndhH, ndhG, ndhJ, ndhK, ndhI* |
| Transcription and translation related genes | Transcription | *rpoA, rpoB, rpoC2, rpoC1^a^* |
|  | Ribosomal proteins | *rps2, rps3, rps4, rps7^c^ (×2), rps8, rps11, rps12^c^ (×2), rps14, rps15^b^, rps16^a^, rps18, rps19, rpl2^a, c^ (×2), rpl14, rpl16^a^, rpl20, rpl23^c^ (×2), rpl33, rpl36* |
| RNA genes | Ribosomal RNA | *rrn16^c^ (×2), rrn23^c^ (×2), rrn4.5^c^ (×2), rrn5^c^ (×2)* |
|  | Transfer RNA | *trnH-GUG, trnK-UUU^a^, trnQ-UUG, trnS-GCU, trnS-UGA, trnS-GGA, trnG-GCC^a^, trnR-UCU, trnR-ACG^c^ (×2), trnC-GCA, trnD-GUC, trnY-GUA, trnE-UUC, trnT-UGU, trnfM-CAU, trnL-CAA^c^ (×2), trnL-UAA^a^, trnL-UAG, trnF-GAA, trnV-GAC^c^ (×2), trnV-UAC^a^, trnM-CAU, trnW-CCA, trnP-UGG, trnI-CAU^c^ (×2), trnI-GAU^a, c^ (×2), trnA-UGC^a, c^ (×2), trnN-GUU^c^ (×2)* |
| Other genes | RNA processing  Carbon metabolism  Fatty acid synthesis  Proteolysis  Conserved open reading frames | *matK*  *cemA*  *accD*  *clpP^b^*  *ycf1^c^ (×2), ycf15^c^ (×2), ycf2^c^ (×2), ycf3^b^, ycf4* |

Note: a indicates that the gene contains only one intron; b indicates that the gene contains two introns; c indicates that the gene is located in the repeat region.

Table S6. ASV identification results of mixed sample experiment

| Primer name | Repeat | ASV | Experimental mixture | | | | | | | Identification result | Identity (%) |
| --- | --- | --- | --- | --- | --- | --- | --- | --- | --- | --- | --- |
|  |  |  | M1 | M2 | M3 | M4 | M5 | M6 | M7 |  |  |
| CS-*ycf1b* | Repeat1 | yASV1 | 23,530 | 1,291,962 | 409,234 | 687,526 | 74,576 | 48,068 | 216 | *P. veitchii* | 100% |
|  |  | yASV2 | 410 | 96,178 | 110,706 | 418,534 | 145,430 | 410,698 | 24,792 | *P. lactiflora* | 100% |
|  |  | yASV3 | 1,926 | 97,790 | 32,226 | 60,418 | 9,278 | 6,384 | 142 | *P. lactiflora* | 99.50% |
|  |  | yASV4 | 480 | 23,818 | 7,068 | 9,078 | 1,444 | 1,040 | 4 | *P. veitchii* | 99.50% |
|  |  | yASV5 | 148 | 9,886 | 3,520 | 5,446 | 518 | 430 | 0 | *P. veitchii* | 99.50% |
|  |  | yASV6 | 76 | 2,218 | 998 | 1,258 | 72 | 98 | 0 | *P. lactiflora* | 98.90% |
|  | Repeat2 | yASV1 | 19,306 | 1,035,324 | 428,940 | 653,890 | 84,360 | 58,900 | 332 | *P. veitchii* | 100% |
|  |  | yASV2 | 318 | 78,960 | 114,168 | 396,166 | 158,634 | 470,070 | 28,126 | *P. lactiflora* | 100% |
|  |  | yASV3 | 1,620 | 79,690 | 33,262 | 58,914 | 10,554 | 7,280 | 166 | *P. lactiflora* | 99.50% |
|  |  | yASV4 | 382 | 20,012 | 8,036 | 9,082 | 1,800 | 1,164 | 0 | *P. veitchii* | 99.50% |
|  |  | yASV5 | 172 | 8,088 | 3,700 | 5,016 | 330 | 472 | 0 | *P. veitchii* | 99.50% |
|  |  | yASV6 | 40 | 2,002 | 906 | 1234 | 110 | 136 | 0 | *P. lactiflora* | 98.90% |
|  | Repeat3 | yASV1 | 23,178 | 1,636,252 | 467,192 | 830,598 | 76,852 | 49,924 | 248 | *P. veitchii* | 100.00% |
|  |  | yASV2 | 436 | 127,262 | 124,004 | 505,642 | 145,112 | 424,674 | 23,810 | *P. lactiflora* | 100.00% |
|  |  | yASV3 | 1,872 | 129,372 | 35,960 | 77,380 | 9,492 | 6,936 | 174 | *P. lactiflora* | 99.50% |
|  |  | yASV4 | 494 | 31,990 | 7,950 | 11,546 | 1,434 | 968 | 6 | *P. veitchii* | 99.50% |
|  |  | yASV5 | 136 | 12,992 | 4,028 | 6,424 | 504 | 446 | 0 | *P. veitchii* | 99.50% |
|  |  | yASV6 | 68 | 3,042 | 1,012 | 1,570 | 120 | 142 | 0 | *P. lactiflora* | 98.90% |
| CS*-ndhF* | Repeat1 | nASV1 | 358 | 722 | 328 | 45,286 | 178,282 | 69,266 | 143,370 | *P. lactiflora* | 100% |
|  |  | nASV2 | 38,462 | 8,844 | 1,660 | 79,156 | 87,682 | 6,666 | 1,234 | *P. veitchii* | 100% |
|  |  | nASV3 | 5,596 | 1,194 | 224 | 12,088 | 16,364 | 1,366 | 390 | *P. veitchii* | 99.50% |
|  |  | nASV4 | 0 | 96 | 30 | 864 | 994 | 88 | 26 | *P. veitchii* | 99.10% |
|  |  | nASV5 | 0 | 90 | 16 | 532 | 706 | 38 | 10 | *P. veitchii* | 99.50% |
|  | Repeat2 | nASV1 | 354 | 694 | 548 | 54,868 | 204,196 | 84,560 | 152,350 | *P. lactiflora* | 100% |
|  |  | nASV2 | 53,808 | 12,376 | 2,976 | 103,608 | 101,712 | 7,094 | 1,192 | *P. veitchii* | 100% |
|  |  | nASV3 | 6,732 | 1,550 | 334 | 14,196 | 17,428 | 1,402 | 342 | *P. veitchii* | 99.50% |
|  |  | nASV4 | 0 | 132 | 30 | 1,084 | 1,164 | 74 | 20 | *P. veitchii* | 99.10% |
|  |  | nASV5 | 0 | 100 | 22 | 676 | 738 | 76 | 6 | *P. veitchii* | 99.50% |
|  | Repeat3 | nASV1 | 268 | 500 | 274 | 43,032 | 206,232 | 68,866 | 158,006 | *P. lactiflora* | 100.00% |
|  |  | nASV2 | 31,486 | 7,228 | 1,178 | 75,760 | 100,940 | 6,368 | 1,316 | *P. veitchii* | 100.00% |
|  |  | nASV3 | 4198 | 1,036 | 130 | 11,772 | 19,092 | 1,274 | 392 | *P. veitchii* | 99.50% |
|  |  | nASV4 | 0 | 82 | 12 | 834 | 1,214 | 78 | 20 | *P. veitchii* | 99.10% |
|  |  | nASV5 | 0 | 76 | 6 | 582 | 868 | 52 | 14 | *P. veitchii* | 99.50% |

Note: The high-quality reads were clustered into amplicon sequence variants (ASV) based on 100% identity. The figures represented the total number of read counts found in libraries.

# Supplementary Figures


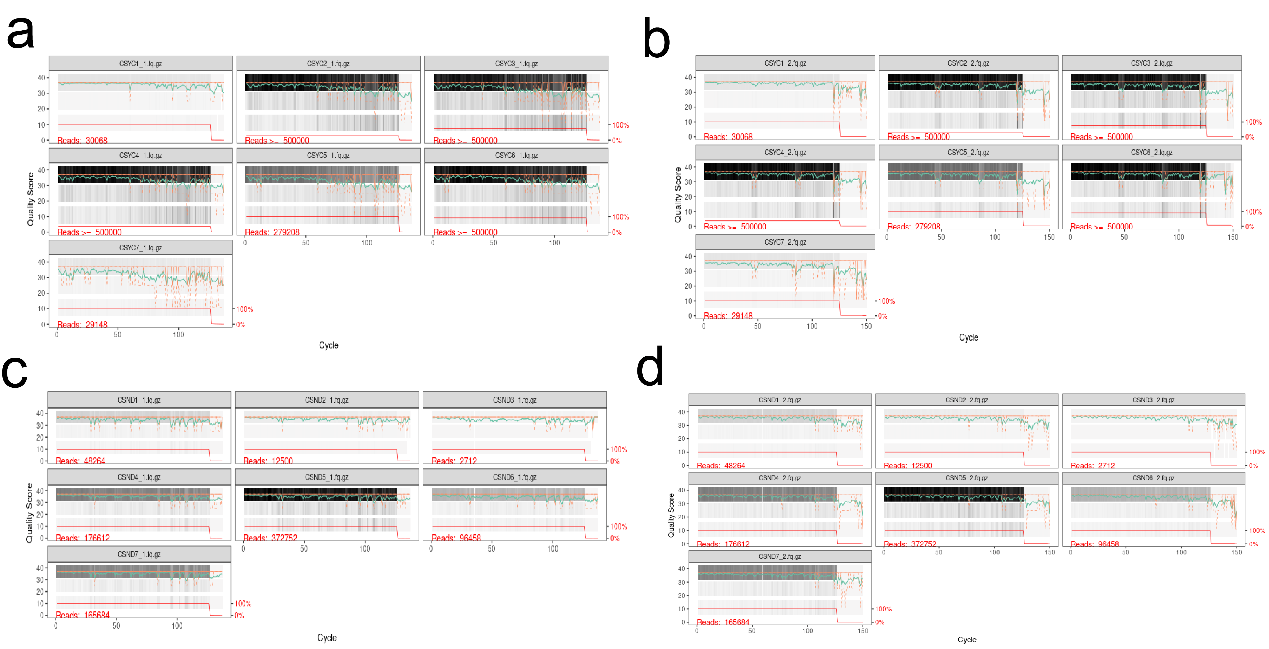


Figure 1-1 The quality distribution of Forward and Reverse bases of repeat1.

Note: The graph was obtained based on the frequency distribution of base quality and was implemented by the DADA2 package. The median quality score at each position was indicated by the green line, the quartiles of the quality score were indicated by the orange line, and the red line represented the proportion of the sequence length extended to that position. We performed quality control on the forward and reverse sequences of amplicon of the stwo pairs of primers, and found that the base quality decreased severely when the sequence length was greater than 120 bp, so we removed this part of the sequence. The software parameters were set as: maxEE = c (2,2), minLen =110, truncLen = c (120,120).

Note: (a) primer CS-ycf1b (Forward); (b) primer CS-ycf1b (Reverse); (c) primer CS-ndhF (Forward); (d) primer CS-ndhF (Reverse)


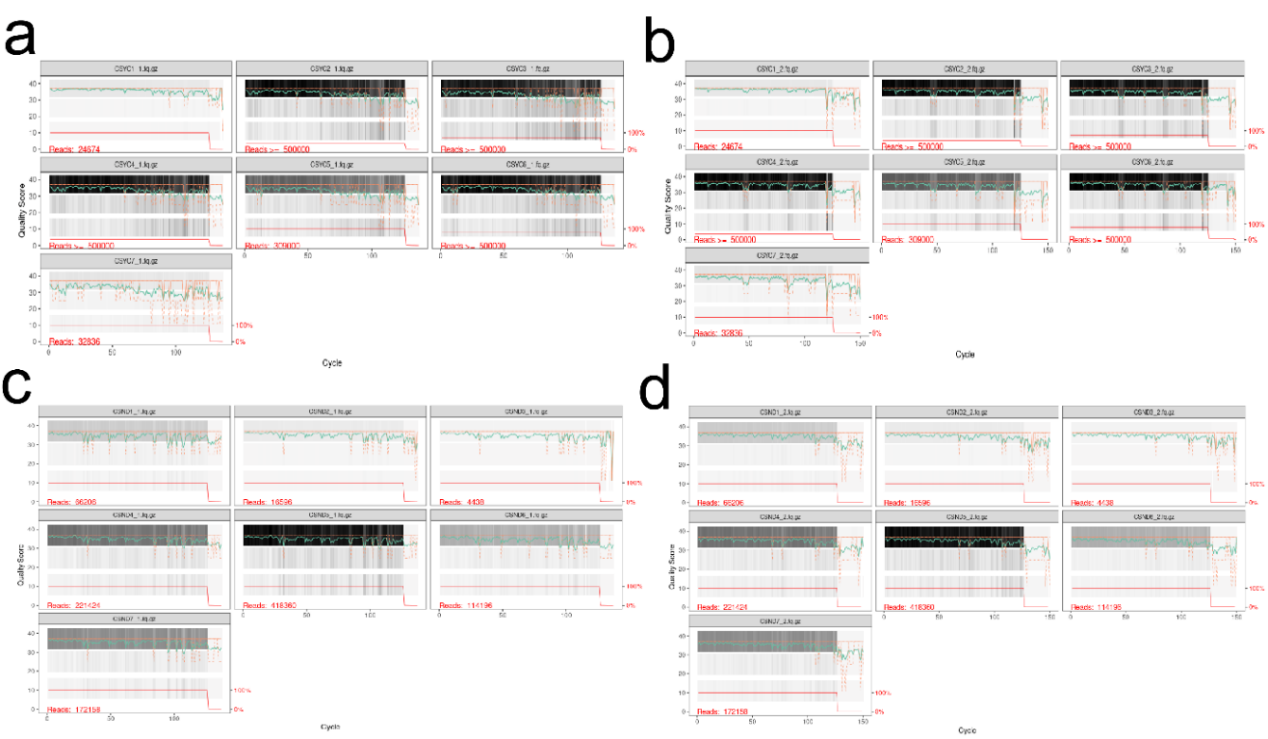


Figure 1-2 The quality distribution of forward and reverse bases of repeat2.

Note: (a) primer CS-ycf1b (Forward); (b) primer CS-ycf1b (Reverse); (c) primer CS-ndhF (Forward); (d) primer CS-ndhF (Reverse).


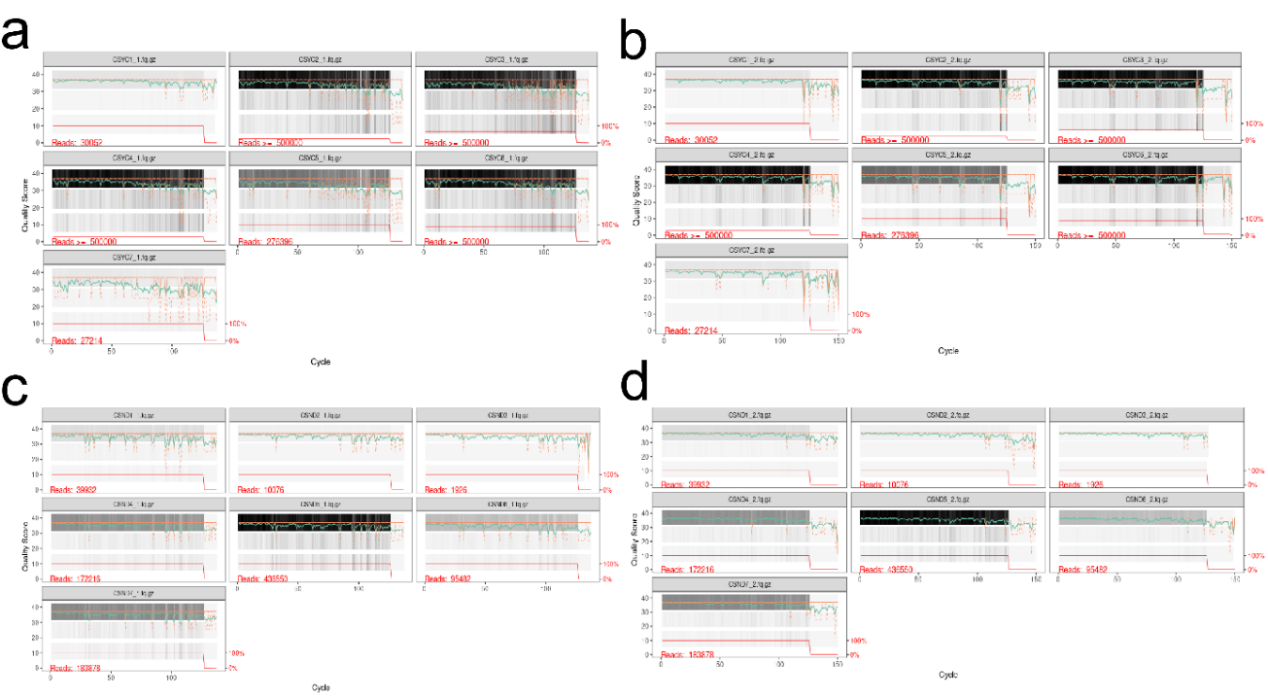


Figure 1-3The quality distribution of forward and reverse bases of repeat3.

(a) primer CS-ycf1b (Forward); (b) primer CS-ycf1b (Reverse); (c) primer CS-ndhF (Forward); (d) primer CS-ndhF (Reverse).


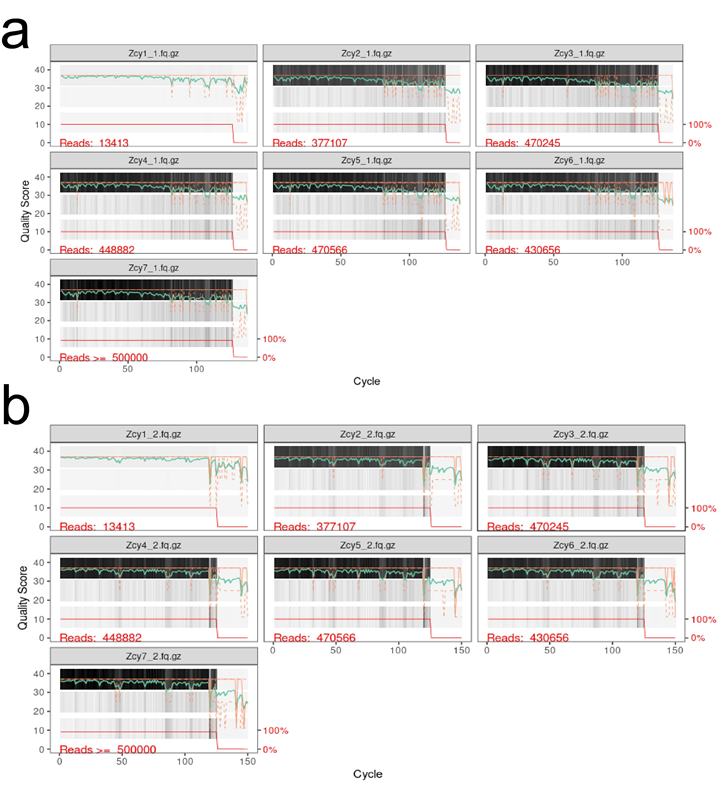


Figure 1-4 The quality distribution of Forward and Reverse bases of the CS-ycf1b amplified product in the Chinese patent medicine.

Note: (a) Forward; (b) Reverse.

**
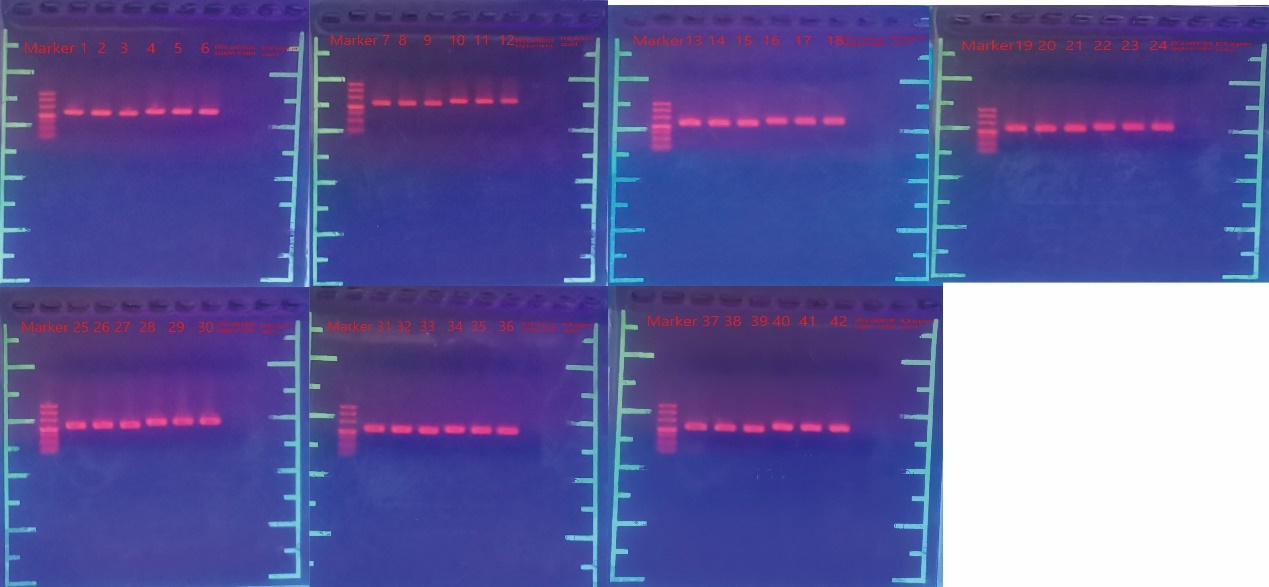
**

Figure 2 The gel electrophoresis diagram of the PCR products of the mixed sample experiment.

Note: 1-6 are the PCR products of mixture1, 7-12 are the PCR products of mixture2, 13-18 are the PCR products of mixture3, 19-24 are the PCR products of mixture4, and 25-30 are the PCR products of mixture5 Products, 31-36 are the PCR products of mixture6, and 37-42 are the PCR products of mixture7.

**
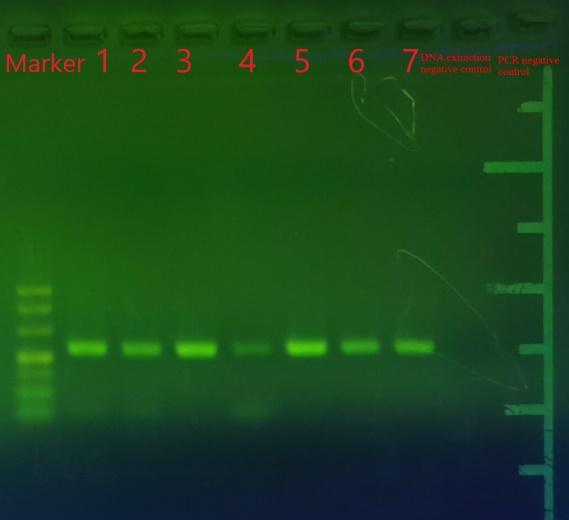
**

Figure 3 Gel electrophoresis diagram of PCR products of Chinese patent medicines.
